# Oral complications of radiotherapy in the head and neck

**DOI:** 10.1016/S1808-8694(15)31029-6

**Published:** 2015-10-19

**Authors:** Bruno Correia Jham, Addah Regina da Silva Freire

**Affiliations:** a M.Sc. in Stomatology, Post-Graduate student.; b PhD in Biology and Oral-Dental Pathology, Assistant Professor at Minas Gerais Federal University Dental School. Federal University of Minas Gerais, Dental School

**Keywords:** Radiotherapy, Oral complications, Head and neck cancer

## Abstract

Radiotherapy is a treatment modality largely used for head and neck malignancies. However, high doses of radiation in large areas, including the oral cavity, maxilla, mandible and salivary glands may result in several undesired reactions. Mucositis, candidosis, disgeusia, radiation caries, osteoradionecrosis, soft tissue necrosis and xerostomia are some of radiotherapy's complications.

**Aim:**

The aim of this study is to briefly review the side effects that may be seen in the oral cavity during or after radiotherapy treatment in the head and neck region.

**Basic method used:**

Review of relevant literature.

**Study design:**

Literature review.

**Results:**

Radiotherapy is still associated with several side effects, significantly affecting patients’ quality of life.

**Conclusions:**

A multidisciplinary treatment, including physicians, dentists, speech therapits, nutritionists, and psychologists, is the best alternative to minimize, or even prevent such reactions.

## INTRODUCTION

Every year, about 870,000 new cases of malignant tumors on the upper airways and digestive passages are diagnosed in the world.^1^ In developing countries, only neck cancer rates are higher than the incidence rates of these malignancies.^2^ The choice treatment for these malignancies is surgery, associated or not to radiotherapy. Surgery and radiotherapy are the treatment options for the localized or regional disease.^3^, ^4^

Many patients with head and neck cancer are submitted to high doses of radiotherapy in large areas including oral cavity, maxilla, mandible and salivary glands. Despite having the advantage of preserving the tissue structure, radiotherapy causes many adverse reactions in the oral cavity.^5^ Since radiotherapy-induced oral complications cause high morbidity and a decrease in quality of life, the aim of this review is to tackle the main adverse oral effects caused by radiotherapy.

### Biological aspects of radiotherapy

Radiotherapy is a treatment option for malign tumors whose therapeutic agent is ionizing radiation, that is to say, the type of radiation that promotes ionization in the area in which it is applied, making it electrically unstable. Ionizing radiations are divided into the corpuscular and electromagnetic ones. Corpuscular radiations are represented by electrons, protons and neutrons; electromagnetic radiations are called photons, being represented by X rays and by gamma rays. In the clinical practice, most radiotherapy treatments are done through the use of photons.^6^

Ionizing radiations act on the nuclear DNA leading to death or loss of its reproductive capacity. Since DNA content duplicates during mitosis, those cells with a high degree of mitotic activity are more radiosensitive than those with low mitotic rate. Radiation action can be direct or indirect. On the direct action, the DNA molecule is cleaved, interfering in the duplication process. On the indirect effect, water is dissociated into its two elements, H+ and OH; the latter reacts with the basis of DNA, interfering in the duplication process. Since water represents most part of cell content, the indirect effect is proportionally more important than the direct one.^7^

Due to the fact of being in a continuous multiplying process, malignant cells can suffer the radiation effects. However, the multiplying ability varies according to cell type. Thus, there is a radiosensitivity scale both for tumor and normal cells. Embryonic malignancies and lymphomas are radiosensitive tumors, whereas carcinomas are moderately radiosensitive.^8^

In order to express the amount of absorbed radiation by the tissues, an international unit, rad (radiation absorbed dose) was initially proposed, that is to say, the difference between the applied radiation and that which went through the tissues. Recently, this unit was replaced by Gray, defined as 1 joule per kilogram. Gy is short for Gray, thus: 1 Gy = 100 cGy =100 rad.^9^, ^10^

Radiotherapy can be administered in short duration schemes up to extremely long schemes, lasting for several weeks. The justification for applications in small daily fractions is based on radiobiology “5 Rs”: reoxygenation, redistribution, recruitment, repopulation and regeneration.^6^ Most patients on radiotherapy receive a total dose of 50-70 Gy as curative dose. These doses are fractioned during a period of 5-7 weeks, once a day, 5 days a week, with a daily dose of approximately 2Gy. On the concomitant treatment, 45c Gy are used on the pre-operative stage and 55-60 Gy on the post-operative.^11^

### Oral complications of radiotherapy

Adverse reactions to radiotherapy will depend on the volume and area being irradiated, on the total dose, on the fractioning, on the age, on the patient's clinical conditions and on the associated treatments. A small increase on tumor dosage is enough for a significant increase on the complications incidence. Acute reactions happen during the treatment and most of the time, they are reversible. Late complications are normally irreversible, leading to permanent incapability and to a worsening of quality of life^5^, and they vary on intensity, being normally classified into mild, moderate and severe.^7^

Many head and neck cancer patients are submitted to high doses of radiotherapy on large areas of radiation including the oral cavity, maxilla, mandible and salivary glands. Thus, anti-cancer therapy is associated with several adverse reactions. These reactions can occur in an acute stage (during or at the weeks right after treatment) or in a chronic stage (months or years after radiotherapy). The severity of acute oral complications will depend on the inclusion degree of these structures on the radiated area.^5^, ^12^

### Mucositis

Mucositis is defined as a mucosal irritation.^5^ Anti-neoplastic-therapy-induced mucositis is a significant adverse reaction that may interfere on the radiotherapy process altering the tumor local control and therefore, the patient's survival. Mucositis is believed to occur in four stages (inflammatory/vascular, epithelial, ulcerative/bacteriologic and healing). The most used scale to measure oral mucositis is the one by the WHO, which classifies mucositis into four degrees. Degree 0 is when there are no signs or symptoms. Degree 1 is when the mucosa is erythematose and painful. Degree 2 is characterized by ulcers, and the patient can eat normally. Degree 3 is when the patient has ulcers and can only drink fluids. Finally, degree 4 is when the patient cannot eat or drink.^13^

Due to oral mucosa damages, patients will complain of pain, what may lead to the need of using painkillers during treatment. The pain intensifies whenever the patient tries to eat or drink.^5^ Mucositis is even worse when chemotherapy is used in association with radiotherapy in cancer treatment.^14^

### Candidosis

Radiated patients are more prone to developing oral infections caused by fungi and bacterias.^15^ Studies have showed that patients submitted to radiotherapy have a higher number of microbian species, such as Lactobacillus spp., Streptococcos aureus and Candida albicans.^9^ Oral candidosis is a common infection in patients being treated for upper airways and digestive tract malignancies. Colonization of oral mucosa can be found in as many as 93% of these patients, whereas Candida infection can be found in 17-29% of patients submitted to radiotherapy. The increased risk for oral candidosis is likely to be the result of the drop in salivary flow as a consequence of radiotherapy.^16^, ^17^ Besides, a possible explanation for a higher predisposition of irradiated patients to candidosis is a reduced phagocytic activity of salivary granulocytes against these micro-organisms.^18^

Clinically, candidosis can be seen both in its pseudomembranous and erythematous forms. The latter can be of difficult diagnosis, and it may be confused with irradiation induced mucositis. Patients complain more of pain and / or burning sensation.^16^, ^17^

Ramirez-Amador et al.^17^ verified that Candida prevalence in patients increased from 43% in the first visit to the doctor's office to 62% during radiotherapy and finally to 75% during post-radiotherapy control visits. In the study by Redding et al.^19^, 73% of patients analyzed showed colonization by Candida, whereas 27% of them had the infection. The study by Grotz et al.^20^ analyzed colonization by Candida in irradiated patients. They verified that the maximum colonization rate happened six months after radiotherapy, and 12 months after radiotherapy the values went back to be lower than normal.

Several studies have already analyzed which Candida species were involved in colonization and infection of radiated patients. Previous studies^16^, ^17^ showed that Candida albicans was the most prevalent micro-organism. However, other species had been identified recently. C. glabrata e C. krusei micro-organisms had already been seen in patients submitted to radiotherapy.^21^ Recent studies found a relation between oral candidosis and C. dubliniensis species. In this study, the authors suggest that the species C. albicans and C. dubliniensis probably act together in the infections that affect radiated patients.^22^, ^23^ Besides, it is known that the non-albicans species distribution varies according to geographical location. Thus, in North America, the predominant species is C. glabrata. Whereas a study done in Brazil showed that the predominant species is C. tropicalis.^24^

### Dysgeusia

Dysgeusia affects patients from the second or third week of radiotherapy onwards, and it may last for several weeks or even months. It occurs because the taste buds are radiosensitive, with the degeneration of their normal histological architecture. The increase of salivary flow viscosity and the saliva biochemical alteration creates a mechanical barrier of saliva which makes it difficult the physical contact between the tongue and foodstuff. The recovery until reaching almost normal levels generally takes place around 60 to 120 days after the end of the radiation. Studies show that dysgeusia is a complaint by approximately 70% of patients submitted to radiotherapy, also implying in the loss of appetite and weight, being the most uncomfortable complication for most radiated patients.^5^, ^14^, ^25^

### Radiation caries

Even patients who had not experienced tooth decay for some time, may develop radiation caries when submitted to radiotherapy.^26^ The main factor for the development of such injuries is the decrease of saliva amount and its qualitative alterations^27^ Besides, radiation has a direct effect on teeth, making them more susceptible to decalcification.^26^

### Osteoradionecrosis

Osteoradionecrosis (ORN) is a bone ischemic necrosis caused by radiation, being one of the most serious consequences of radiotherapy, causing pain as well as possible substantial loss of bone structure^5^, ^28^ Due to anti-cancer therapy, bone cells and the vascularization of bone tissue may suffer irreversible injuries.^26^ ORN may occur spontaneously or more commonly, after trauma (generally dental extractions). In 95% of cases ORN is associated with soft tissue necrosis and subsequent bone exposure.^28^ Mandibles are more affected than maxillas and patients with their natural teeth have greater chances of developing ORN. Spontaneous bone exposure occurs approximately one year after finishing radiotherapy and the risk of developing this complication remains indefinitely.^26^ Besides, studies show that approximately 60% of patients complain of pain, ranging from mild pain, controlled with drugs, to extremely painful conditions. However, the presence of these symptoms does not appear to be related to the extension of the process. ORN may also result in edema, suppuration and pathological fractures, which may occur in 15% of patients, always experienced together with pain.^28^

### Soft tissue necrosis

Another possible consequence of radiotherapy is soft tissue necrosis, which may be defined as an ulcer located in the radiated tissue, without the presence of residual malignancy. The occurrence of soft tissue necrosis is related to dose, time and volume of the radiated gland, when the brachytherapy is used, the risk is higher. Soft tissue necrosis is a normally painful condition and good oral hygiene together with the use of painkillers and often times, antibiotics, are necessary to manage the condition. Since ulcerations are often seen on the tumor primary site, regular evaluations are necessary until the necrosis retreats, therefore excluding the possibility of recurrence.^26^ Besides, soft tissues may suffer fibrosis after radiotherapy, becoming pale, thin and without flexibility. When fibrosis affects chewing musculature (temporal, masseter and pterygoid muscles) trismus can happen. In the most serious cases, trismus may interfere with eating and dental care.^5^

### Xerostomia

Xerostomia, or “dry mouth”, can result from some diseases or it can be an adverse reaction to some drugs. 29 Among radiated patients in the head and neck area, it is one of the most frequent complains.^30^ Chencharick and Mossman^25^ noticed that 80% of radiated patients complain of xerostomia. However, the relation between the individual perception of dry mouth and the real values of salivary flow havr not yet been completely defined.^31^ In some situations, there is a co-relation between reduced salivary flow and xerostomia complaint.^32^ However, in many cases there is not a relation between xerostomia and objective findings of salivary gland dysfunction - that is to say, patients without alterations of salivary flow may complain of mouth dryness. Patients with xerostomia complain of oral discomfort, taste loss, speech and swallowing difficulties.^33^ Saliva also suffers qualitative alterations resulting from radiotherapy with decrease of amylase activity, buffer capacity and pH, with consequent acidification. There are also alterations of several electrolytes such as calcium, potassium, sodium and phosphate^26^, ^27^, ^28^, ^29^, ^30^, ^31^, ^32^ Thus, individuals who were radiated are more susceptible to periodontal disease, rampant tooth decay and oral infections by fungus and bacteria.^15^

Xerostomia treatment can be done through the use of mechanic/taste stimulants, saliva substitutes or systemic agents.^37^, ^38^ Alternative methods, such as acupuncture, had also been mentioned as treatment options for xerostomia.^39^ Generally speaking, stimulants and saliva substitutes only reduce xerostomia, without altering salivary flow. On the other hand, systemic agents besides reducing xerostomia, also decrease oral problems associated with salivary glands hypofunction, through the increase of salivary flow. Thus, the treatment of choice for radiotherapy-induced xerostomia, should be through the use of systemic agents, and pilocarpine is the most studied one among them. Besides, studies show that systemic agents, such as pilocarpine, are more effective when used during radiotherapy.^34^, ^40^ This has also been recently showed for betanechol, when the drug used concomitantly with radiotherapy is able to increase salivary flow at rest, right after the end of the radiotherapy treatment, besides decreasing the subjective complaint of dry mouth.^41^

## CONCLUSIONS

Radiotherapy has been widely used in treating malignant lesions on the head and neck, with improvement in patient survival rates. However, this therapy is still associated with several adverse reactions that affect patient quality of life significantly, and may even affect the progress of the treatment. Taking into account that the occurrence of head and neck cancer rates are probably going to be the same as last decades, it is extremely important that health professionals are familiarized with the complications that may result from anti-neoplastic treatments. Multidisciplinary treatment, including medical team, dental surgeons, speech therapists, nutritionists and psychologists, is the best option in order to minimize or even prevent such complications.
